# Supplementary figures and images for: Chalcones identify cTXNPx as a potential antileishmanial drug target
Source: PLoS Negl Trop Dis. 2021 Nov 15;15(11):e0009951. doi: 10.1371/journal.pntd.0009951 (PMC8664226; doi:10.1371/journal.pntd.0009951)

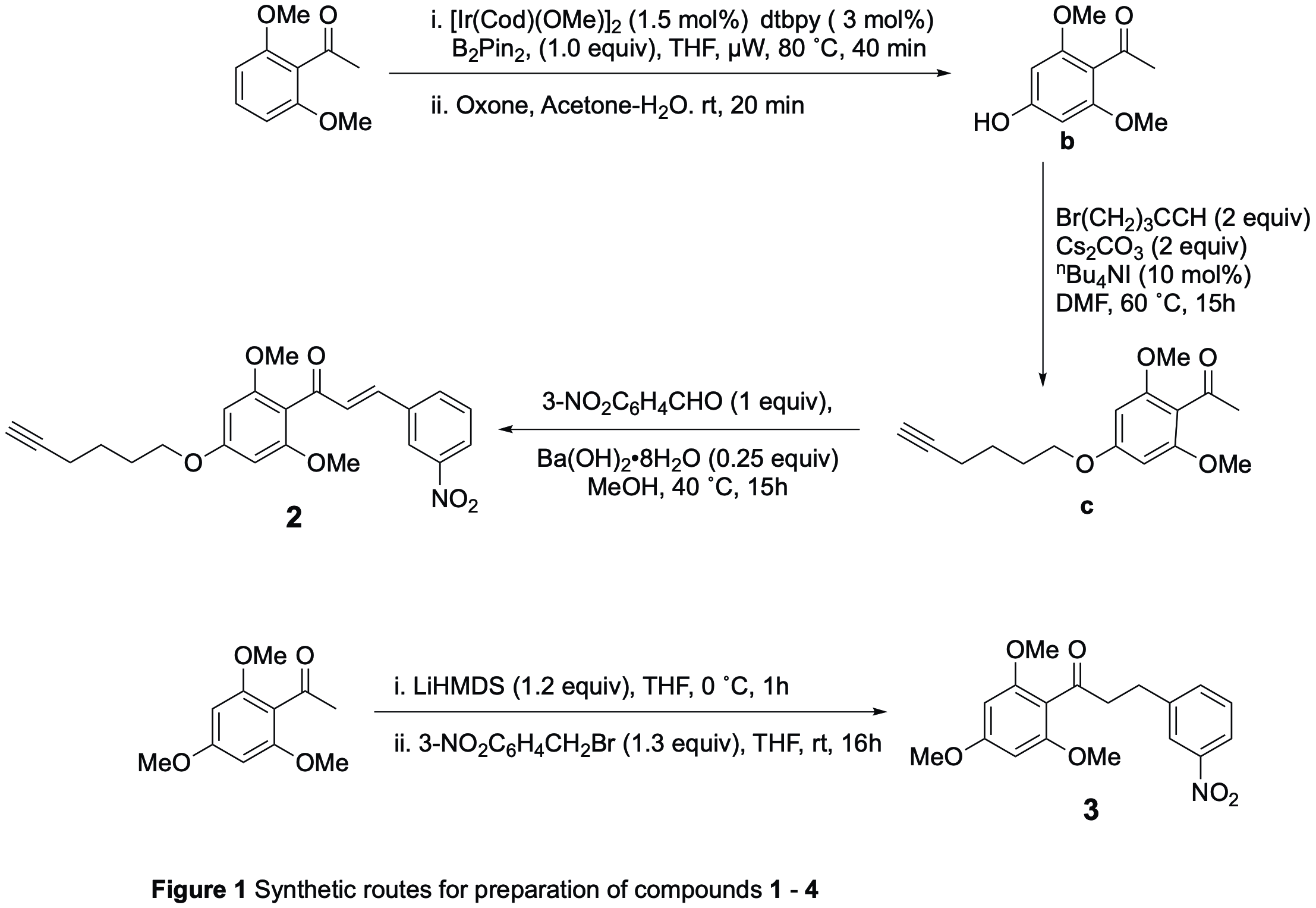

Supplement: S1 Fig — (TIF) [file pntd.0009951.s001.tif]

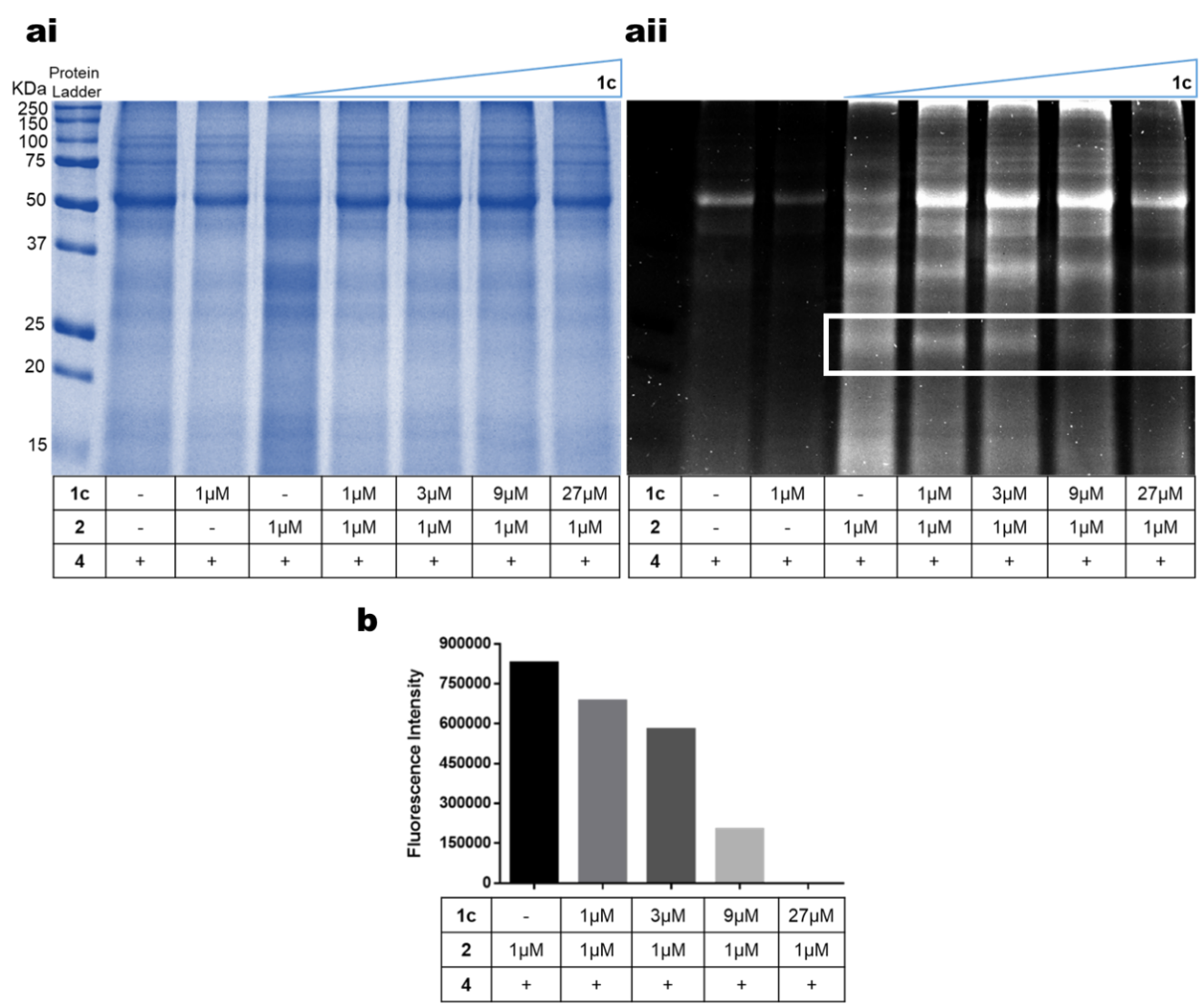

Supplement: S2 Fig — (ai) SDS-PAGE of all tested conditions stained with comassie blue. (aii) Fluorescence image of all tested conditions. (TIF) [file pntd.0009951.s002.tif]

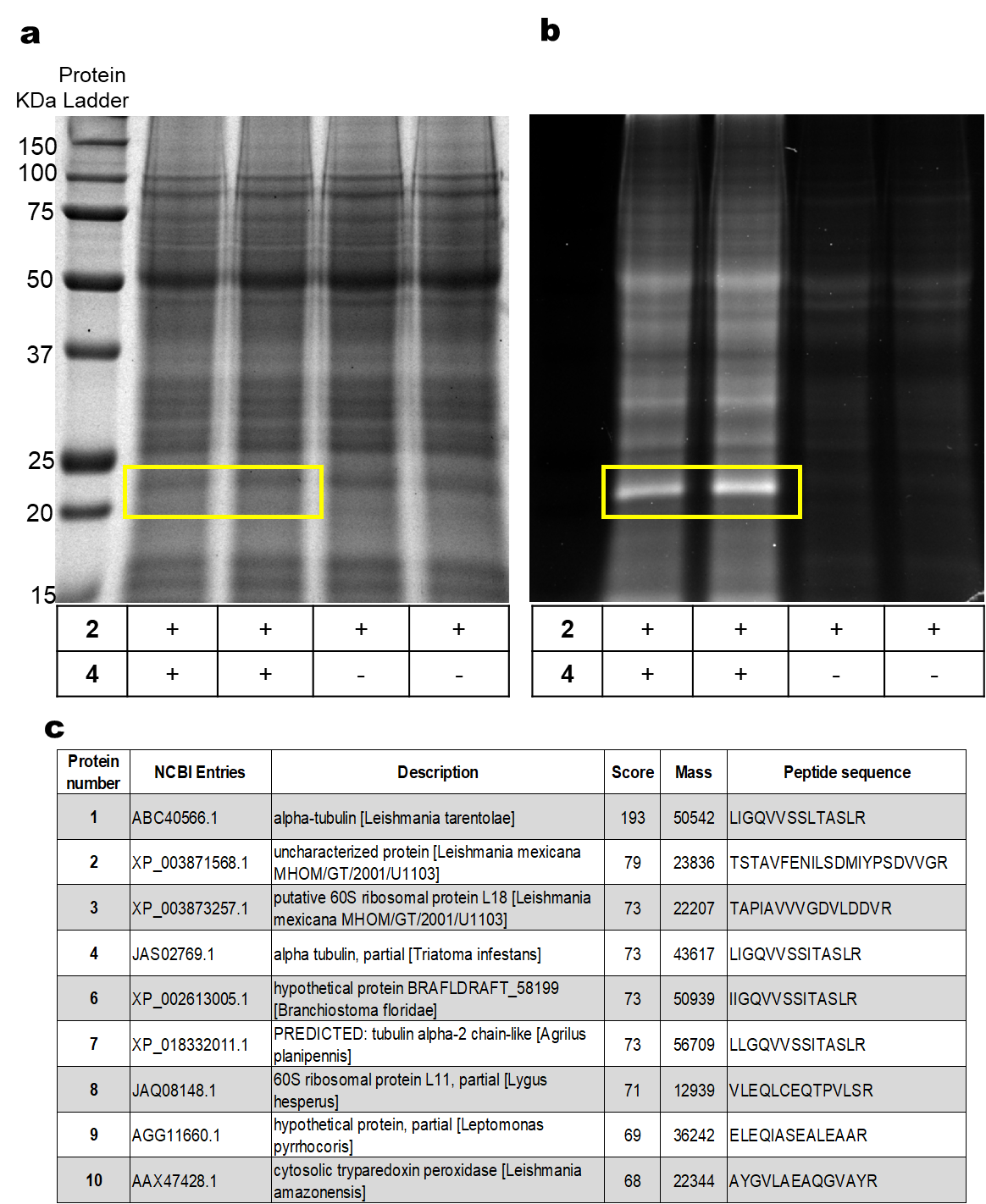

Supplement: S3 Fig — (a) SDS-PAGE stained with Coomassie blue. (b) In-gel fluorescence image. Yellow rectangles- Protein bands excised for mass spectrometry analysis. (c) Proteins candidates identified from the band. (TIF) [file pntd.0009951.s003.tif]

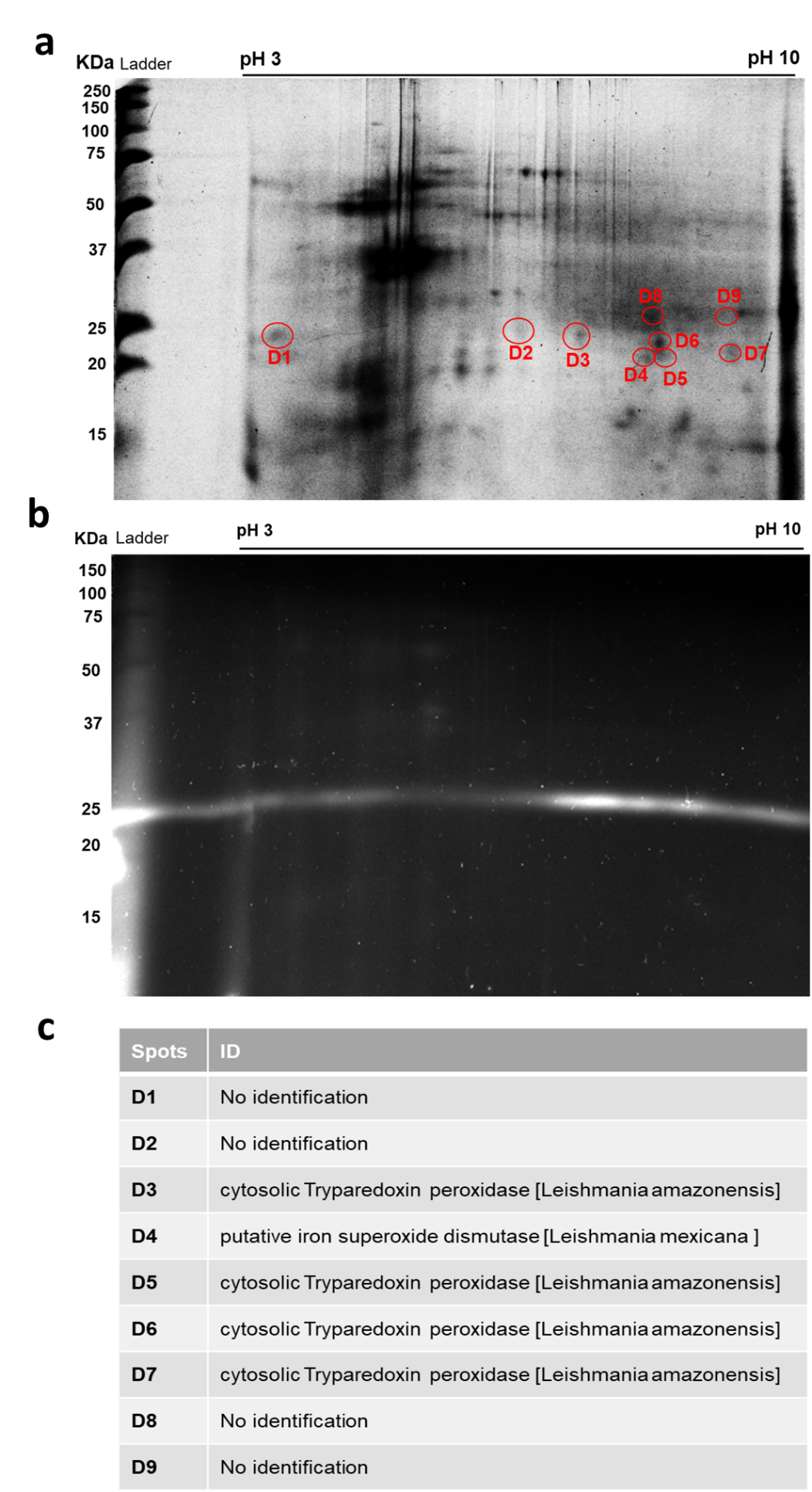

Supplement: S4 Fig — (a) Map of the dots excised from the 2D gel stained with comassie blue and submitted to mass spectrometry analysis. (b) In-gel fluorescence detection image. (c) Proteins candidates identified for each 2D dot. (TIF) [file pntd.0009951.s004.tif]

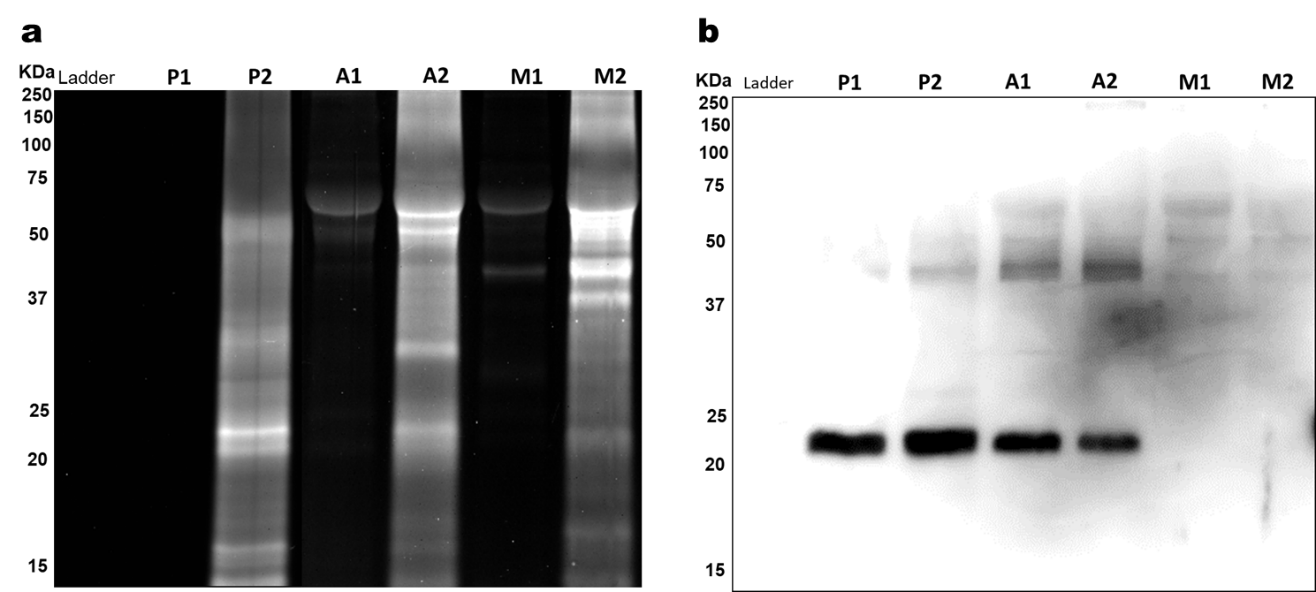

Supplement: S5 Fig — Proteins from promastigotes (Pro), isolated intracellular amastigotes (Ama), and macrophage (Mac) lysates that had been pre-incubated with 2 (5 μM) and linked to 4 (5 μM) prior to fluorescence revelation. Alternatively, the run lysates were revealed with mouse anti-cTXNPx antibody followed by anti-Mouse IgG-HRP (anti- cTXNPx). a. In-gel fluorescence detection image. b. Immunoblotting analysis with anti-cTXNPx. Proteins from promastigotes (P1), amastigotes (A1) and macrophages (M1) without 2. Proteins from promastigotes (P2), amastigotes (A2) and macrophages (M2) pre-incubated with 2. (TIF) [file pntd.0009951.s005.tif]

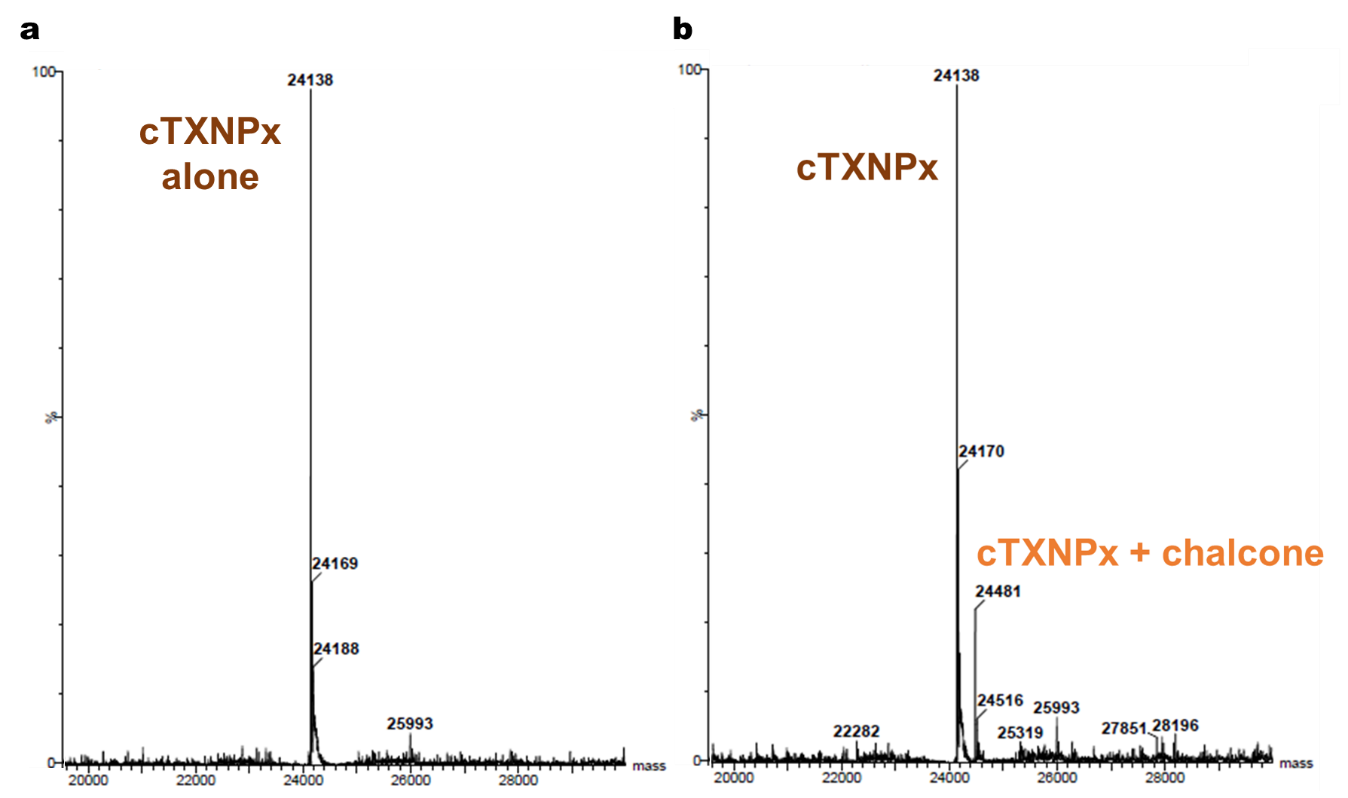

Supplement: S6 Fig — Purified proteins (0.6 mg/ mL) were incubated or not with compound 1c (1:1) for 30 min and their molecular weight analysed by Mass Spectrometry. (a) cTXNPx of L. major molecular weight [M-Met requires 24138 Da]. (b) cTXNPx of L. major with 1 [M-Met+343 requires 24481 Da]. (TIF) [file pntd.0009951.s006.tif]

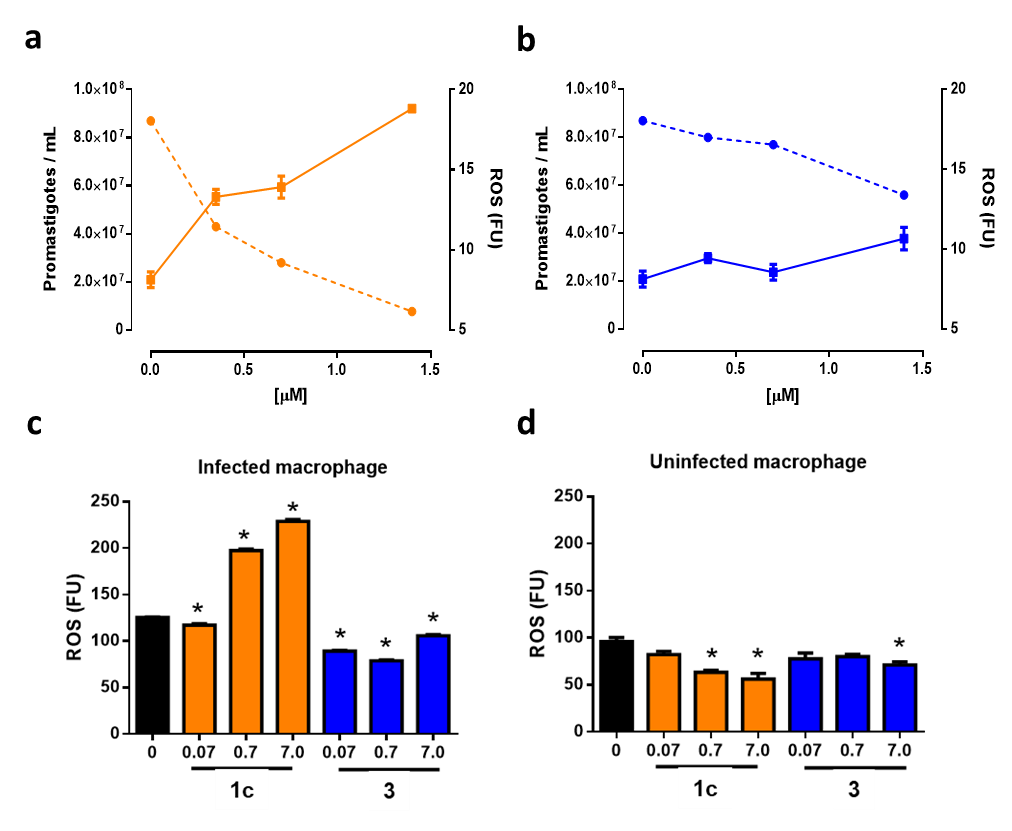

Supplement: S7 Fig — L. amazonensis promastigotes (1 x 10^6^/mL) were incubated with compounds 1c or 3 at the indicated concentrations for 48 h, during which the numbers of cells were recorded. Then, 4 x 10^5^ cells were transferred to black 96 well plates for fluorimetric assessment of ROS production with H2DCFDA (20 μM) for 30 min. (a-b) Parasite killing (dotted lines) and ROS production (continuous lines) induced by 1c (a) 3 (b). BMDM (1 x 10^5^/well) infected or not with L. amazonensis promastigotes (10:1) for 72 h on 96 well-plate, then cells were incubated with 1c or 3 (0.07, 0.7 and 7.0 μM) for 1 h at 37°C and ROS production measured as described for promastigotes. (c) ROS production on infected macrophages. (d) ROS production on uninfected macrophages. Means ± SD (n = 3). * p<0.05 in relation to untreated cells. (TIF) [file pntd.0009951.s007.tif]

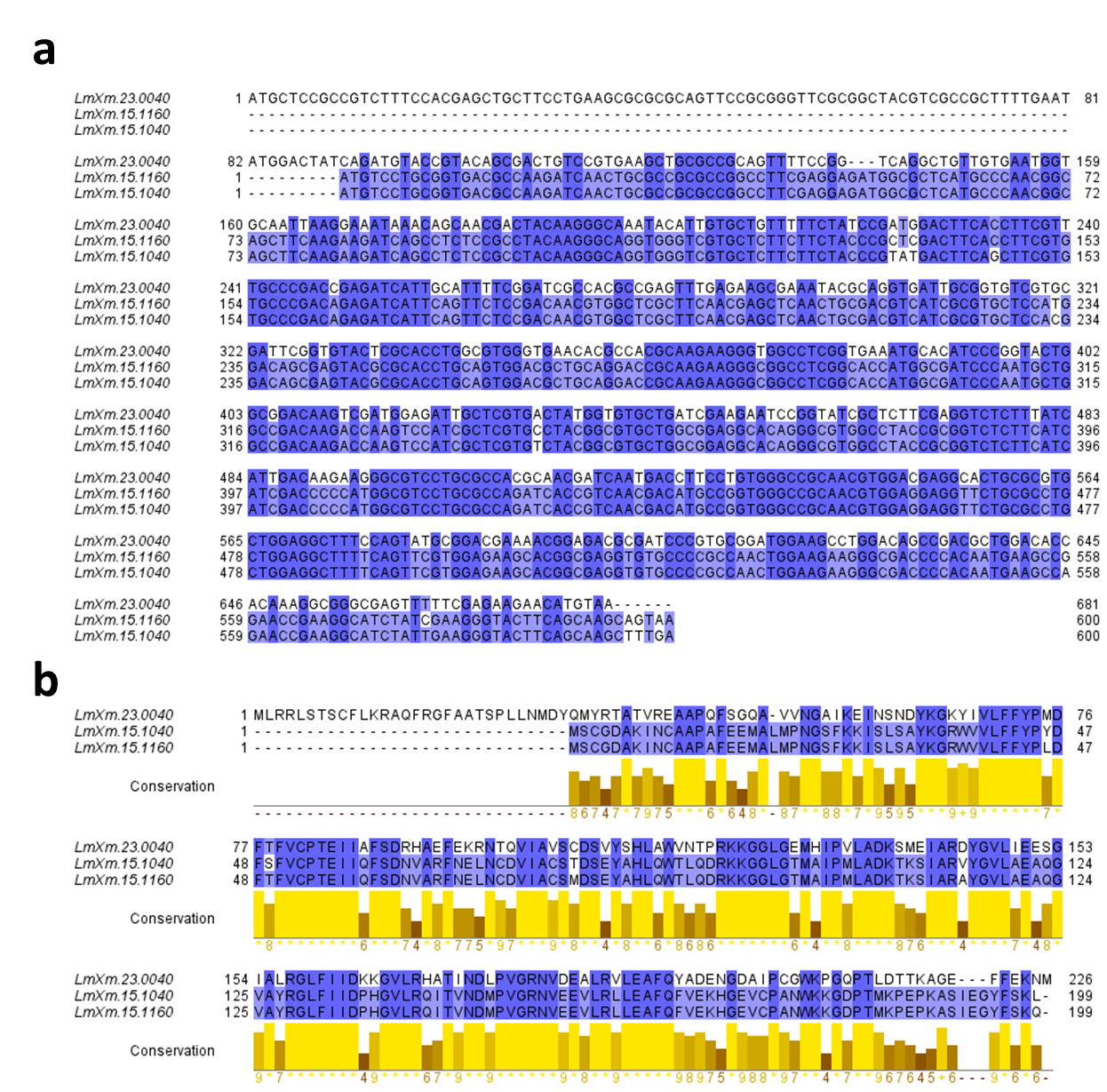

Supplement: S8 Fig — Gene and protein sequences of mitochondrial (LmxM.23.0040) and cytosolic (LmxM.15.1040 and LmxM.15.1160) TXNPx were obtained from https://tritrypdb.org website and aligned using Jalview software (2.11.1.3). (a) Gene sequence alignment (b) Protein sequence alignment. (TIF) [file pntd.0009951.s008.tif]

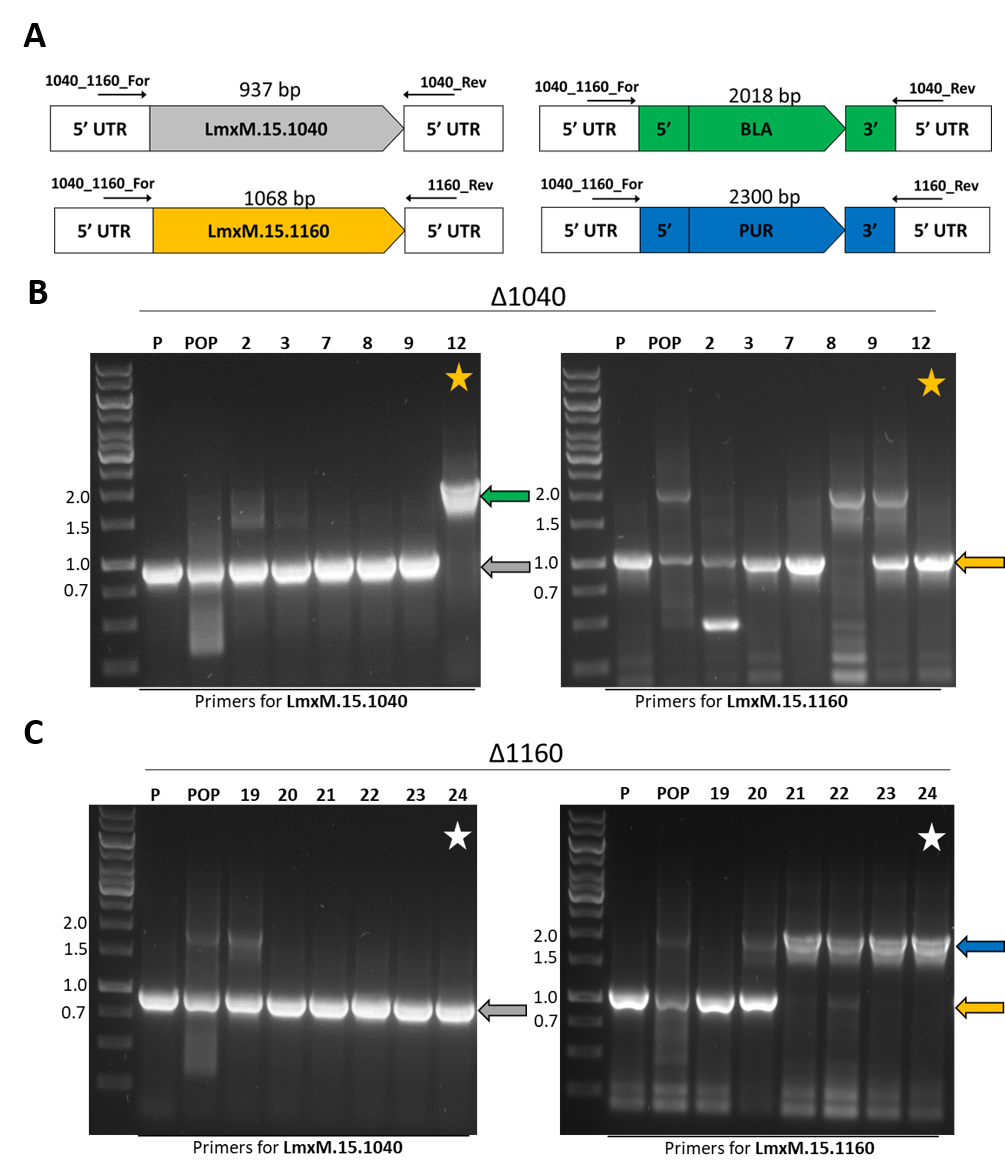

Supplement: S9 Fig — (a) Diagrams showing PCR strategy for assessing presence of gene copies for LmxM.15.1040 and LmxM.15.1160 and their replacement with the blasticidin [BLA] or puromycin [PUR] cassette. (b) PCR of clonal lines following transfection with sgRNA targeting LmxM.15.1040 gene with BLA as the repair cassette. Yellow star highlights clonal double KO of LmxM.15.1040 (left; green arrow BLA and grey arrow LmxM.15.1040 CDS) and the retention of LmxM.15.1160 (right; yellow arrow LmxM.15.1160 CDS). (c) PCR of clonal lines following transfection with sgRNA targeting LmxM.15.1160 with PUR as the repair cassette. White star highlights clonal double KO of LmxM.15.1160 (right; blue arrow PUR and yellow arrow LmxM.15.1160 CDS) and the retention of LmxM.15.1040 (grey arrow LmxM.15.1040 CDS). P—positive control. Pop—population. (TIF) [file pntd.0009951.s009.tif]
