# Supplementary material for: Chalcones identify cTXNPx as a potential antileishmanial drug target
Source: PLoS Negl Trop Dis. 2021 Nov 15;15(11):e0009951. doi: 10.1371/journal.pntd.0009951 (PMC8664226; doi:10.1371/journal.pntd.0009951)

**Chalcones Identify cTXNPx as a new Potential Antileishmanial Drug Target**

Synthetic procedures

**Chemistry general procedures**. IR spectra were acquired using a Perkin-Elmer Spectrum 1000 FT-IR Spectrometer equipped with an ATR module, and absorption maxima ν_max_ were reported as wavenumbers (cm^−1^). Nuclear Magnetic Resonance (NMR) spectra were acquired on Varian VNMRS 700 (^1^H at 700 MHz, ^13^C at 176 MHz) or Varian VNMRS 600 (^1^H at 600 MHz, ^13^C at 151 MHz). Chemical shifts are reported in ppm (δ). High resolution mass spectra (HRMS) were obtained using Waters LCT Premier XE with an electrospray ionization (ESI) source.

**1-(4’-(hex-5”-yn-1”-yloxy)-2’,6’-dimethoxyphenyl)-3-(3”’-nitrophenyl)prop-2-en-1-one 2**

2,6-dimethoxyacetophenone (0.18 g, 1.0 mmol) was dissolved in dry THF (1 mL), and added to a mixture of B_2_pin_2_ (0.25 g, 1.0 mmol, 1.0 equiv), [Ir(OMe)(cod)]_2_ (9.9 mg, 1.5 mol %), and 4,4′- di-tert-butyl-2,2′-dipyridine (8.0 mg, 3 mol %) in THF (1 mL) in a sealed microwave vessel under nitrogen. The reaction was then heated in a microwave reactor at 80 °C for 40 minutes. Then the reaction mixture was filtered through silica gel and the solid residue washed with DCM (2 x 5mL). The combined filtrate was then concentrated under reduced pressure to afford the boronate ester (**a**) which was used directly in the next step with no further purification. The crude boronate ester (**a)** was dissolved in acetone (2 mL) in a round flask and oxone (1.1 equiv) in water (2 mL) was added and the reaction was stirred for 20 minutes at RT, then sodium bisulfite (1.1 equiv) was added to quench the reaction. After acetone evaporation, the mixture was extracted with DCM (3 x 7mL) and the combined organics concentrated under vacuum. The crude product was dissolved in EtOAc (15 ml) and extracted by acid-base extraction (NaOH 1M and HCl 3M). The crude product was purified by flash column chromatography (EtOAc/hexane = 1:1) to afford 2,6-dimethoxy-4-hydroxyacetophenone **b** (61%). All data was in accordance with the literature[^1^](#_ENREF_1).

2,6-dimethoxy-4-hydroxyacetophenone (**b**) (1.00 g, 5.10 mmol) and 5-bromohexyne (1.63 g, 10.20 mmol) was dissolved in DMF (10 mL), then Cs_2_CO_3_ (3.32 g, 10.20 mmol) and Bu_4_NI (0.19 g, 0.51 mmol) was added. The reaction was stirred overnight at 60°C, and the mixture was then washed with water (2 x 10 mL) and the product extracted with DCM (2 x 10 mL). The organic layer was washed with water (2 x 10 mL), Brine (1 x 10 mL) and dried over MgSO_4._ The product was concentrated under vacuum and purified by flash column chromatography (hexane/EtOAc = 8:2) to afford 2,6-dimethoxy-4-(hex-5’-ynyloxy)acetophenone (**c**).

A mixture of the acetophenone (**c**) (74 mg, 0.27 mmol), 3-nitrobenzaldehyde (40 mg, 0.27 mmol) and Ba(OH)_2_.8H_2_O (21 mg, 0.07 mmol) in MeOH (5 mL) was stirred overnight at 40°C. After solvent evaporation, the product was extracted with EtOAc (3 x 10 mL) and the combined extracts washed with HCl - 1M (2 x 10mL), dried over MgSO_4_ and concentrated under vacuum. The crude product was a yellow oil that was purified by flash column chromatography (hexane/EtOAc = 4:1), to afford the desired title chalcone **2**. ∂­_H_ (599 MHz, CDCl_3_) 8.34 (m, 2”’-*H*, 1H), 8.20 (m, 4”’-*H*, 1H), 7.83 (m 6”’-*H*, 1H), 7.55 (m, 5”’-*H*, 1H), 7.43 (d, J= 16.0 Hz, 3-*H*, 1H), 7.06 (d, J= 16.0 Hz, 2-*H*, 1H), 6.16 (s, 2’,4’-*H*, 2H), 4.05 (t, J= 6.3 Hz, 1”-*H*_2_, 2H), 3.78 (s, OC*H*_3_, 6H), 2.30 (m, 4”-*H*_2_, 2H), 2.03 – 1.85 (m, 2”-*H*_2_,6”-*H*, 3H), 1.79 (m, 3”-*H*_2_, 2H); ∂_C_ (151 MHz, CDCl_3_) 193.1, 162.4, 159.3, 148.8, 140.2, 137.1, 133.9, 131.6, 123.0, 124.4, 122.8, 111.5, 91.4, 84.1, 68.9, 67.7, 56.1, 28.3, 25.1, 18.3; ν_max_ (ATR) 3291 (C≡C-H), 2939 (C-H Alkane), 1653 (C=O) cm^-1^; HRMS (ES^+^): found [MH]+ 410.1585; C_23_H_25_NO_6_, requires *M,* 410.1604.

**3-(3’-nitrophenyl)-1-(2”,4”,6”-trimethoxyphenyl)propan-1-one 3**

A solution of 1-(2,4,6-trimethoxyphenyl)ethanone (210 mg, 1.0 mmol) in anhydrous THF (3 mL) at 0 ºC was treated with LiHMDS solution in anhydrous THF (1.2 mL of a 1M solution in THF, 1.2 mmol). The reaction mixture was then stirred for 1h at 0°C. Benzyl bromide (281 mg, 1.3 mmol) in THF (2 mL) was added as a solution and the resulting mixture was stirred at RT overnight (o/n). Saturated ammonium chloride solution (2 mL) was added to quench the reaction followed by DCM (20 mL). The aqueous phase was washed with DCM (3 x 10 mL) and combined organic phase was dried over MgSO_4_ and solvent removed *in vacuo.* The compound was purified by reverse phase chromatography (5 to 100% MeCN in H_2_O) to afford 3-(3’-nitrophenyl)-1-(2”,4”,6”-trimethoxyphenyl)propan-1-one **3** (60 mg, 0.17 mmol). ∂_H_ (700 MHz, CDCl_3_) 8.07 (m, 2’-*H*, 1H), 8.03 (m, 4’-*H*, 1H), 7.58 (m, 6’-*H*, 1H), 7.42 (m, 5’-*H*, 1H), 6.08 (s, 3”,5”-*H*, 2H), 3.81 (s, 4”-OC*H*_3_, 3H), 3.74 (s, 2”,6”-OC*H*_3_, 6H), 3.13 – 3.12 (m, 2-*H*_2_, 3-*H*_2_, 4H); ∂_C_ (176 MHz, CDCl_3_) 202.5, 162.6, 158.4, 148.4, 143.7, 135.2, 129.2, 123.3, 121.2, 113.1, 90.8, 55.9, 55.6, 45.3, 29.6; ν_max_ (ATR) (ATR) 1696 (C=O), 1525, 1347 (N=O) cm^-1^; HRMS (ES^+^): found [M]+ 346.1298; for C_18_H_20_NO_6_ requires M, 346.1291.

^1^H- and ^13^C-NMR spectra of chalcone probe **2**.


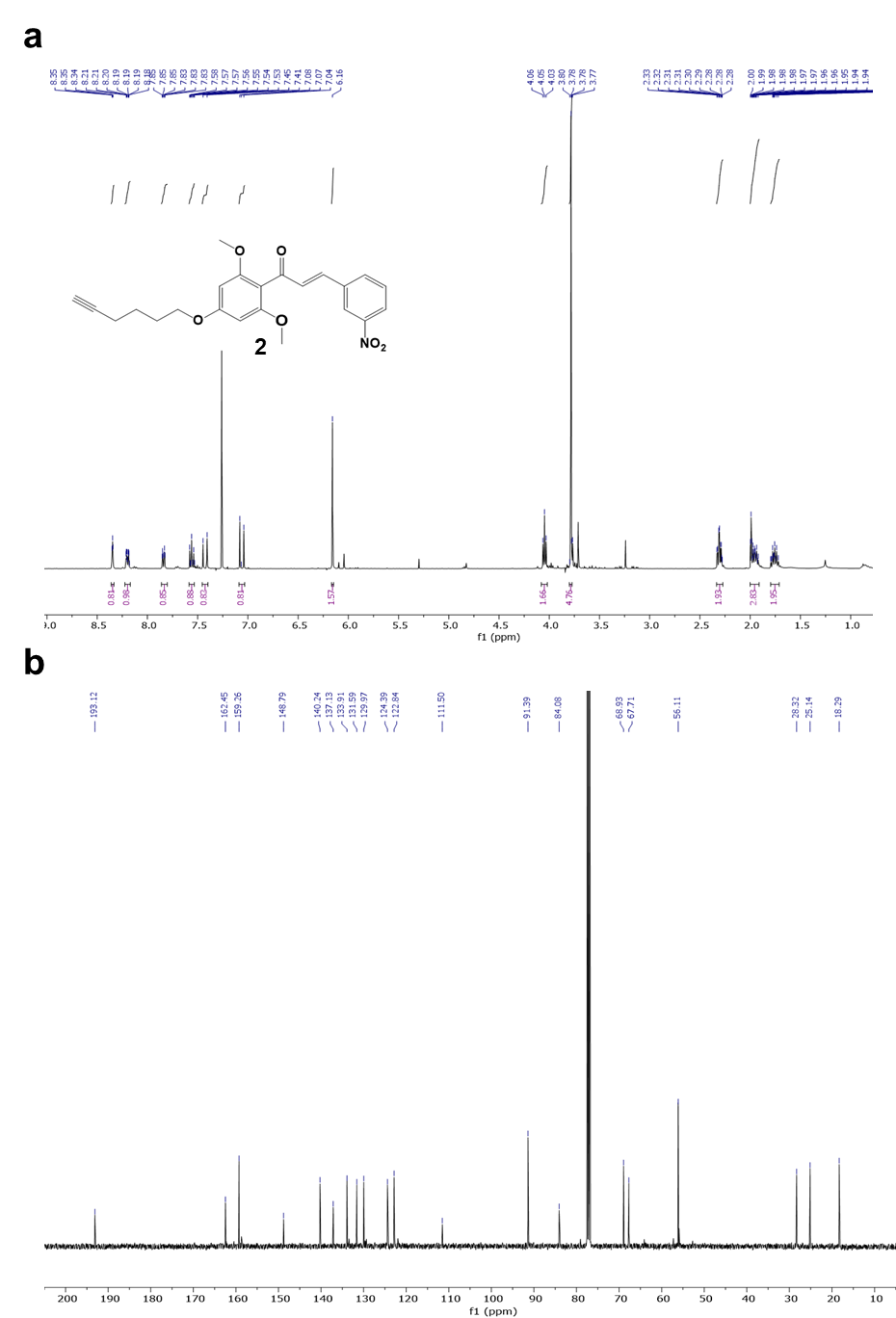


^1^H- and ^13^C-NMR spectra of dihydrochalcone **3**


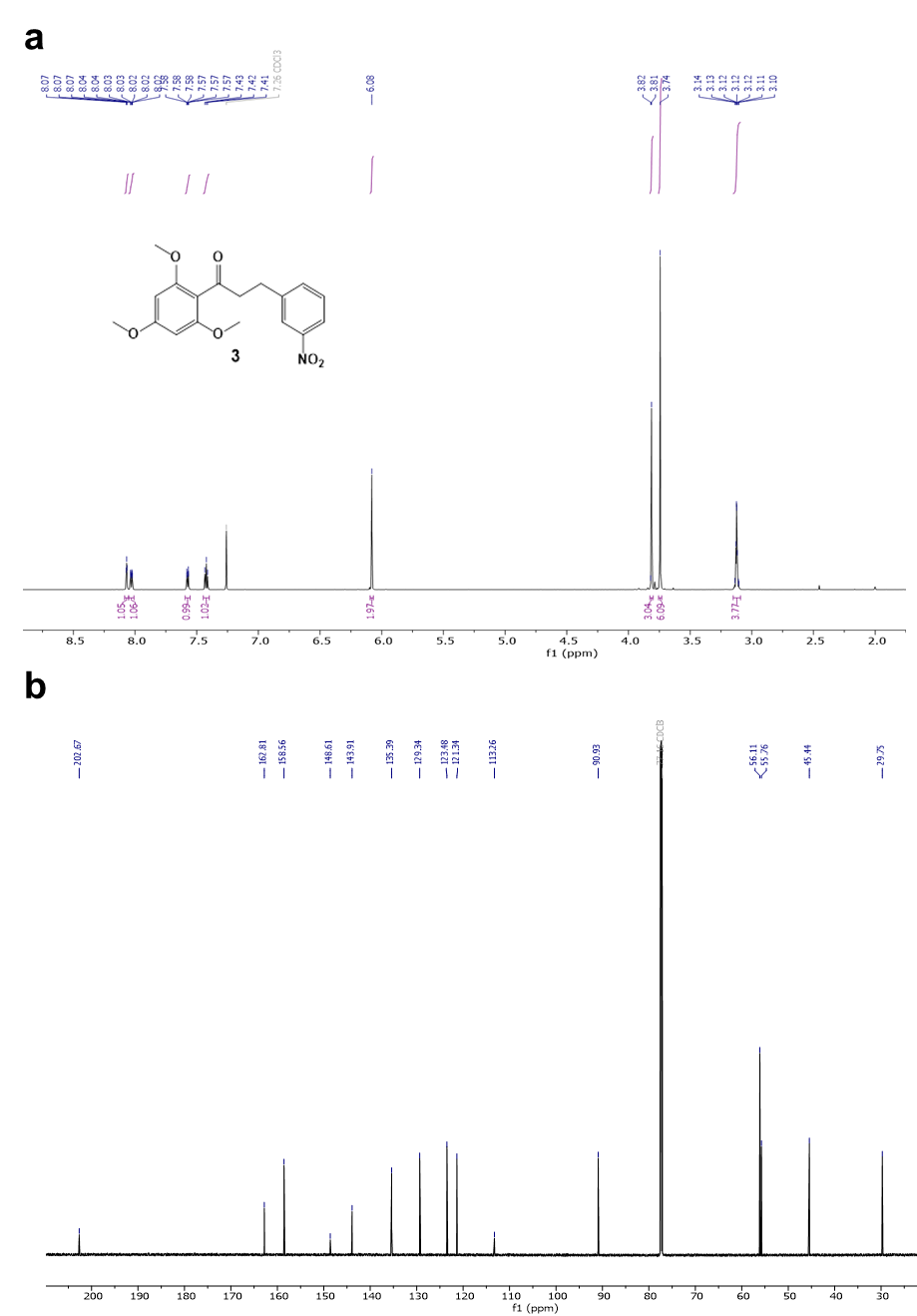

Supplement: S1 Text — (DOCX) [file pntd.0009951.s014.docx]
