# Supplementary material for: Chalcones identify cTXNPx as a potential antileishmanial drug target
Source: PLoS Negl Trop Dis. 2021 Nov 15;15(11):e0009951. doi: 10.1371/journal.pntd.0009951 (PMC8664226; doi:10.1371/journal.pntd.0009951)
Supplement: Attachment — Submitted filename: Response_to_Referees_Escrivani_et_al.docx [file pntd.0009951.s015.docx]

**Authors response to referee’s comments**

We thank the referees for their time in reading and providing their helpful comments that have helped to improve the manuscript.

We have reviewed all the comments made and addressed them indicating the changes made, as described below:

-----------------------------------------------------------------------------------------------

Reviewer #1: The methods described in this study were appropriated and adequately described in the manuscript. The samples size (particularly for in vivo essays) was adequate and answered the hypothesis of the authors. Finally, there is no concern about ethical requirement.
Some minor points:
- In the Anti-promastigote activity, please include the growth phase of parasites used in the assays (page 6, lines 111-120).

Line 115: Promastigotes of *L. amazonensis* (strain MHOM/BR/75/Josefa, 5 x 10^5^/mL), *L. braziliensis* (strain MHOM/BR/75/M2903, 2 x 10^6^/mL) and *L. infantum* (strain MHOM/MA67ITMAP263, 2 x 10^6^/mL) at **the logarithmic phase of growth…**
- Please, indicate the number of mice used for in vivo experiment, using chalcone 1c (page 6, lines 148-160).

Lines 155-156 now reads: “BALB/c mice were **randomly distributed into groups of 5 and** intradermally infected in the right ear pinna with 2 × 10^6^ *L. amazonensis* promastigotes at stationary phase of growth”.

- Table S2, Neo-forward primer is missing in the Table.

The primer name, Neo_For, has been added to supplementary table S4. To clarify the strategy used in this work, we have revised Fig 6A and also added a supplementary S9 Fig with more details of the CRISPR-Cas9 work

Reviewer #2: The authors clearly designed a methodology that attended the main questions regarding the purpose of the work: the elucidation of a mechanism of action of chalcones that results in their antileishmanial effect. The in vivo analyses were made under ethic procedures validated by the ethic committee, as described in the methodology. At least 3 independent experiments were performed and the statistical analysis is proper, allowing to address the objectives. The use of competition assay, ABPP, and Leishmania cTXNPx knockouts supported by docking analysis were very important to confirm the hypothesis that TXNPx is a potential target for chalcones in the treatment of leishmaniasis.

Reviewer #3: Methods are sound and well explained. There is only one points I would like the authors to address: For mice infections and treatment, the authors must add to the method section who many animals were used per group/experiment, how many times the experiments were repeated and which statistical methods were used to decide the number of animals used to achieve statistical significance.

Lines 155-156 now reads: “BALB/c mice were **randomly distributed into groups of 5 and** intradermally infected in the right ear pinna with 2 × 10^6^ *L. amazonensis* promastigotes at stationary phase of growth”.

“Animal sample size was estimated by a power-based method using the following assumptions: α = 0.05, 1 − β = 0.9, and standard deviation 20% of mean for groups.” Lines 162-164.

We also added the new reference Charan et al. 2013 to support our method.
--------------------

Reviewer #1: Results are well presented throughout the manuscript. The figures, in general, are clear and have good and enough quality for the reader.
Some minor points:
- Figure 2A is a Table, so please replace it for a Table in the final version of the manuscript (page 27).

We feel that as originally presented the inclusion of the EC_50_ values as a table within Figure 2 is the most effective way of conveying this information. We note that similar presentations of data can be found in other articles in in PLOSNTD and there is no formal requirement for to exclude tables from figures. Consequently, we kindly request that this table remain as a component of Figure 2.

- Figure 2C, include the standard deviation of the limiting dilution assay (vehicle group) (page 27).

We have increased the thickness of the bar and changed the colour of the column to make sure it is seen. A new figure 2C was added.

- Figures S3-C and S4-C should also be replaced by supplementary Tables.

Figures S3-C and S4-C were converted into Table S1 and S2 respectively.

Reviewer #2: The results are very clear and describe efficiently the analysis plan. The figures are sufficiently clear and well delineated.

Reviewer #3: Probe displacement assay - Are 2 and 4 permeable to life cells? If so, I would suggest to show the dose dependent probe displacement assay in live cells to prove target engagement of cTXNPx by 1c, and for future campaigns for the target. The assay will also allow for further calculations of inhibition as Ki and Kd of the compounds. The assay could be anyway developed for the purified recombinant protein to gain information on the kinetics of association/dissociation of the compound with the target. Although the gel-based assay is informative, it is hardly quantitative and is limited to the target binding.

Chalcone probe 2 is permeable to live cells and has similar anti-promastigote effects in comparison to the chalcone 1c. This data is presented in the table in Figure 2A. Probe 4 is not permeable to live cells. Both compounds are covalent labelling agents which challenges the calculation of Ki and Kd values. We accept the fact that that gel based images are qualitative and, accordingly, have adjusted the text to read as follow:

Lines 984-985 “White rectangle -Decreasing fluorescence with” was replaced by “Qualitative decrease in fluorescence signal by”.

Essentiality - The authors had shown that compound 1c binds to cTXNPx and defend it to be the molecular target responsible for its toxicity to Leishmania. These results go into conflict with the data showing that the genes composing the complex are dispensable in promastigotes. Also, the authors did not provide any evidence of the requirement of the genes to amastigotes. I highly recommend for the mutants to be also investigated as amastigotes in macrophages and/or mice infections.

Whilst cTXNPx KO cell lines can be generated, as seen by the microscopy images, the resultant parasites are severely morphologically compromised. We also note that in the experiments to measure ROS production we have been able to show that the increased ROS production in a macrophage is only observed in chalcone treated infected cells consistent with impaired TXNPx activity in the chalcone treated amastigote form (S7 Fig). Whilst we agree that experiments to evaluate whether or not parasite mutation also interferes with parasite infectivity in macrophages and animals would interesting, this is non-trivial and represents a different study to this work which is focused on identifying molecular targets of chalcones.

--------------------

Reviewer #1: The conclusions of the manuscript are clear and well supported by the results.

Reviewer #2: The authors conclude that the work validates TXNPx as a much-needed drug target for the treatment of leishmaniasis. Really, the current treatment possibilities have limitations, such as high prices, toxicity, and parasitic resistance. An important observation that could be better used to reinforce the importance of this work is that tryparedoxin peroxidases, such as TXNPx, are highly conserved among trypanosomatids, without direct mammalian orthologue. This suggests that chalcones could be a leishmanicidal drug with a low possibility of side effects in mammals.
As commented in the text, leishmaniasis is a disease that impacts millions of people worldwide and has a high economic and social impact. The public health relevance is adressed, considering the problems related to existing drugs and the fact that leishmaniasis are neglected diseases that can be disfiguring or fatal, depending on the clinical form presented.
The authors seem quite sure of the limitations of their analyses, and do not jump to conclusions about their findings.

Reviewer #3: Mode of action proposed - The authors discuss the role of cTXNPx in ROS resistance and suggest its inhibition as the mode of action for 1c, but do not provide any evidence for increase of ROS mediated cell death after treatment. These are well stablished assays that should be performed by the authors for better understanding and support of the mode of action here proposed.

We do not understand this question. In our study we provided strong evidence that treatment with 1c resulted in increased ROS production by both promastigotes and intracellular amastigotes after treatment with 1c. This data is presented in the main text (page 13, line 389 -402) and in the supplementary material (S7 Fig.).

--------------------

Editorial and Data Presentation Modifications?
Use this section for editorial suggestions as well as relatively minor modifications of existing data that would enhance clarity. If the only modifications needed are minor and/or editorial, you may wish to recommend “Minor Revision” or “Accept”.

Reviewer #1: (No Response)

Reviewer #2: Minor issues:
1.      Line #38: “Instituo” – Instituto;

Changed in line 38.

2.      Line #86: The period is missing after “antileishmanials”;

Period was added after “antileishmanials” in line 87.
3.      Line #87: Remove the comma;

Comma was removed from the sentence in line 88.
4.      Lines #92-93: “...which showed strong and selective anti-parasite activity in vitro and in vivo against both CL and VL”. Replace CL and VL with the Leishmania species;

CL and VL were replaced by *Leishmania amazonensis* and *Leishmania infantum* in line 94.
5.      Line #165: cite the permeabilizing agent used;

Sentence in line 168-170 was changed to “PBS, and fixed with 4% of paraformaldehyde and permeabilized with 0.1% saponin in PBS for 5 min at room temperature (RT).”.
6.      Line #309: “Single-variance analysis (ANOVA) and t-test was used...” – Single-variance analysis (ANOVA) and t-test were used...;

Line 318: was changed to were.
7.      Line #318: “Whilst CH8 (1b; Fig 1) showed good activity”. What good activity? Against what? Same for the line #327;

Thank you for this observation, figure numbers were added in this sentence by mistake. Figure numbers were removed and “against *L. amazonensis*” added to address the parasite used on reference #32 in line 333-334.
8.      Line #934: Change Leishmania to Leishmania in italic.

Line Leishmania changed to *Leishmania* in line 963

- Revise the English with a mother tongue specialist.

- In general, the text has to be revised considering spacing of characters, punctuation, and some other spelling details. For example:

. Line #111: “5 x 105/mL” instead of “5 x 105 / mL”;
. Line #114: “72 h” instead of “72h”;
. Line #122: “26 °C” instead of “26° C”;
. Line #155: “of growth [16].” instead of “of growth.[16]”;
. Line #181: “1 h” instead of “1 hr”;
. Line #190: “1 h at room temperature” instead of “1 hr at RT”;
. Line #574: “leishmaniasis” instead of “leishmanaisis” ...

The manuscript was carefully revised to check those details.

Reviewer #3: Line 86 - Missing the period at the end of the phrase.

Period was added after “antileishmanials” in line 87.

Line 148 - Please indicate the number of animals used for mouse infection and treatment/parasite burden. Please provide the statistical method used to determine the number of animals necessary to achieve a reliable assessment. Also, in Fig. 2C provide a figure with results for each mouse and the exact p value obtained for the assay. Also, does the result represent independent assays? Does n=5 mean 5 animals/assay?

Thank you for your point. These information were added to the text as:

“animals were randomly distributed into groups of 5” line 156 and “Animal sample size was estimated by a power-based method using the following assumptions: α = 0.05, 1 − β = 0.9, and standard deviation 20% of mean for groups.” Line 162-164.

We also added the new reference Charan et al. 2013 to support our method.

Line 284 - Please correct prefix (LmxM) of gene ID for LmxM.15.1040 and LmxM.15.1160 (also in other parts of the text, as line 432).

The prefix has been corrected throughout the manuscript.

Line 335 and Figure 2A - The authors mix the use of IC50 and EC50 for cellular based assays in the text and Figure 2A, as well as in other parts of the text in experiments that have a similar read-out. Please consolidate the use of EC50 to report results based on cell death response to compound treatment.

All IC50s have been replaced by EC50.

Line 346 - Please insert space after Fig 3C.

Space inserted after Fig 3C in line 352.

Line 451 - The statement ‘To investigate if the specific cTXNPx enzyme is the chalcone target in cellulo’ must be rethought and rephrased since the method used by the authors only assess the cell viability after treatment, not the target engagement.

We are sorry but we do not understand this question. The various labelling experiments presented in the paper provided compelling evidence that the chalcone does engage with cTXNPx. Taken collectively with the fact that deletion of the enzyme in the parasite raises the EC_50_  value of the chalcone is consistent with inhibition of cTXNPx activity in a cellular context. We accept that the chalcone may have multiple targets within the parasite and have therefore amended the text (Line 536) to read “To investigate if cTXNPx is a chalcone target in cellulo”

--------------------

Summary and General Comments
Use  this section to provide overall comments, discuss strengths/weaknesses of the study, novelty, significance, general execution and scholarship. You may also include additional comments for the author, including concerns about dual publication, research ethics, or publication ethics. If requesting major revision, please articulate the new experiments that are needed.

Reviewer #1: In this manuscript, the authors described the activity in vitro and in vivo of a nitroanalogue (compound NAT22, 1c) against Leishmania species. This compound is a derivative of a natural chalcone with previously reported activity against Leishmania in vitro and in vivo. The authors also described that the cytosolic tryparedoxin peroxidase (cTXNPx) is the main target of this compound, leading to ROS accumulation and parasite death. These findings were confirmed by deletion of cTXNPx genes by CRISPR-Cas9 technology. Finally, molecular docking studies confirmed that the compound 1c is able to bind the putative active site of cTXNPx.
In general, the manuscript is clear and well written; however some points should be considered for publication.

Main points:
- Concerning to the diagnostic PCR for mutant cell lines of cTXNPx genes, did the authors only investigate for LmXm.15.1160 coding sequence, as indicated in the Figure 6A (right panel)? What about the other copy (LmXm.15.1040 gene)? In Table S2, it is indicated that there are specific primers for each one of these genes.

Initially, using specific sgRNAs, LmxM.15.1040 and LmxM.15.1160 were knocked out separately and clonal lines isolated. This was confirmed by gene specific PCR (new S9 Fig). Subsequently, a LmxM.15.1040 and LmxM.15.1160 double knockout was created from one of these clones using another repair cassette (NEO gene). Figure 6A has been revised to give more detail about this approach (line 1015).

- Please, include in the Figure 6A, the PCR product expected for LmXm.15.1040 coding sequence.

Figure 6A was reformulated to clarify our approach (line 1015).

- It is also missing the diagnostic PCR for ORF of cTXNPx genes in each one of mutant cell lines (Δ1040 and Δ1160), using parental line as control. Please include it in the manuscript.

Figure 6A was reformulated to clarify our approach (line 1015).

- Were mutant cell lines (Δ1040 and Δ1160) generated by loss of heterozogosity? These mutant cell lines only have one drug resistance marker. Please specify this point in the manuscript. In the agarose gel for ΔcTXNPx line, it is missing the PCR for blasticidin S deaminase gene.

In these two mutant cell lines (Δ1040 and Δ1160) both gene copies were deleted and replaced by their respective repair cassette (BLA and PUR). This was confirmed by PCR (new S9 Fig). Subsequently a new round of transfection was performed on clonal Δ1160, targeting the remaining LmxM.15.1040 using a NEO repair template. To clarify this Figure 6A has been revised and we have added more details in the methods and results sections to better explain our approach.

- Please, include standard deviation of EC50 values of the mutant cell lines (page 31, Fig. 6D).

Standard deviation was added to EC_50_ values – Fig 6D (Fig 6E) line 1015.

- I would also suggest to present the graphical abstract as a Figure in the manuscript.

The figure in line 1012 is our proposed graphical abstract.

- Line 1012 – Specify TOC.

TOC means Table of Contents image – we have now titled this as a Graphical Abstract to remove any further confusion.

Reviewer #2: Overall, the work was very well designed, clearly written, and presents a novelty regarding the mechanism of action of a promising drug against leishmaniasis. Considering some suggested spelling corrections and the need to reinforce the issue of possible drug selectivity in the treatment of infection in mammals, which reinforces the importance of this work, it is able to be accepted for publication in PLOSNTD.

Reviewer #3: cTXNPx null mutant - I found the way that the diagnostic PCRs were shown misleading, and did not understand well the strategy. The final cell line contains 1 copy of LmxM.15.1040 deleted by BSDr gene, and 1 copy of LmxM.15.1160 by PACr gene? But how did the second copies of both genes were deleted by only NEOr in one go? Please clarify the strategy in the text and include in Fig 6A the agarose gels that show presence/absence of both CDS in each step of the genetic manipulation for better understanding.

Modifications were added in the manuscript to explain better our approach, including:

-Figure 6A was replaced by Figures 6A and 6B.

-A new S9 Figure (Supplementary material).

-Text has been modified in both the methods and results sections as noted above.
